# Supplementary material for: A Microfluidic Concentration Gradient Maker with Tunable Concentration Profiles by Changing Feed Flow Rate Ratios
Source: Micromachines (Basel). 2020 Mar 10;11(3):284. doi: 10.3390/mi11030284 (PMC7142998; doi:10.3390/mi11030284)
Supplement: Supplementary file 1 [file micromachines-11-00284-s001.pdf]

micromachines

Supplementary Materials: A Microfluidic Concentration Gradient Maker with Tunable Concentration Profiles by Changing Feed Flow Rate Ratios

Tao Zhang, Jiyu Meng, Shanshan Li, Chengzhuang Yu, Junwei Li, Chunyang Wei and Shijie Dai

Received: 22 February 2020; Accepted: 8 March 2020; Published: date

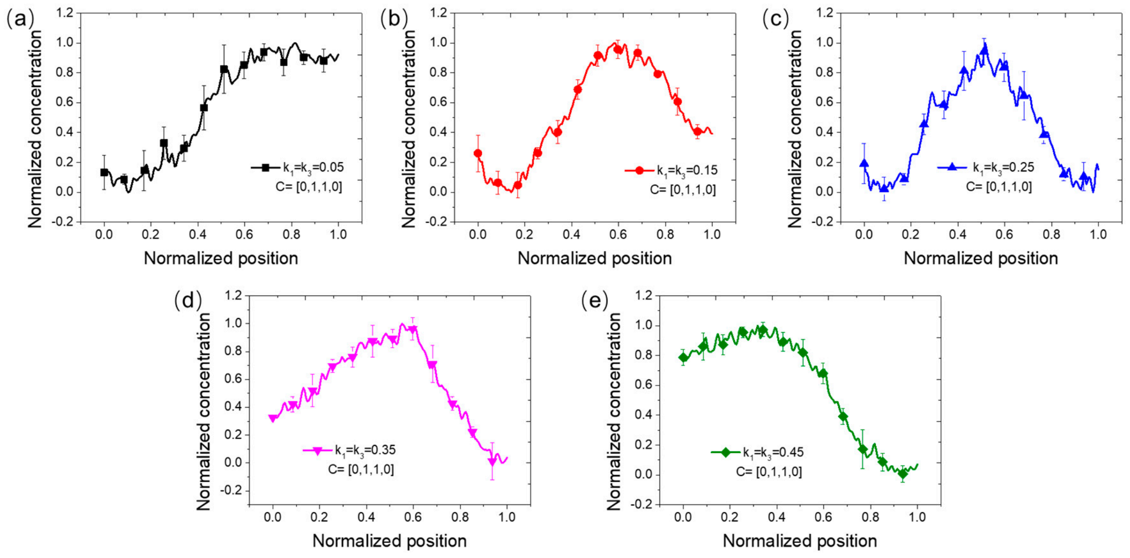

Figure S1. The repeatability of the type IV feed flow rate ratio (FFRR) microfluidic mixer. The average results of five experimental groups (normalized concentration against normalized position) are shown at the outlet of the type IV microfluidic device with the concentration matrix C = (0, 1, 1, 0). (**a**)–(**e**) denote the cases of $k_1 = k_3 = 0.05$; $k_1 = k_3 = 0.15$; $k_1 = k_3 = 0.25$; $k_1 = k_3 = 0.35$; and $k_1 = k_3 = 0.45$, respectively. The maximum error is 15.1% and most of the errors are within 10%.

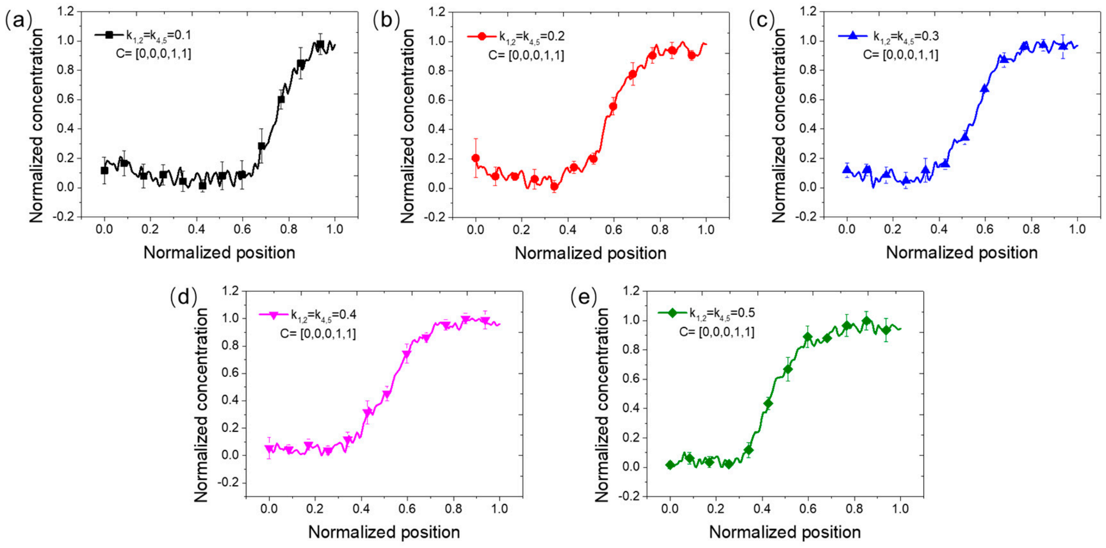

Figure S2. The repeatability of the type V FFRR microfluidic mixer. The average results of five experimental groups (normalized concentration against normalized position) are shown at the outlet of the type V microfluidic device with the concentration matrix C = (0, 0, 0, 1, 1). (**a**)–(**e**) denote the cases of $k_{1,2} = k_{4,5} = 0.1$; $k_{1,2} = k_{4,5} = 0.2$; $k_{1,2} = k_{4,5} = 0.3$; $k_{1,2} = k_{4,5} = 0.4$; and $k_{1,2} = k_{4,5} = 0.5$, respectively. The maximum error is 13.7% and most of the errors are within 10%.
